# Structure-Function Relationship of Inclusion Bodies of a Multimeric Protein

**DOI:** 10.3389/fmicb.2020.00876

**Published:** 2020-05-08

**Authors:** Anupam Singh, Vaibhav Upadhyay, Akansha Singh, Amulya K. Panda

**Affiliations:** Product Development Cell, National Institute of Immunology, New Delhi, India

**Keywords:** inclusion bodies, active inclusion bodies, amyloid content, biological activity, amyloid structure

## Abstract

High level expression of recombinant proteins in bacteria often results in their aggregation into inclusion bodies. Formation of inclusion bodies poses a major bottleneck in high-throughput recovery of recombinant protein. These aggregates have amyloid-like nature and can retain biological activity. Here, effect of expression temperature on the quality of *Escherichia coli* asparaginase II (a tetrameric protein) inclusion bodies was evaluated. Asparaginase was expressed as inclusion bodies at different temperatures. Purified inclusion bodies were checked for biological activities and analyzed for structural properties in order to establish a structure-activity relationship. Presence of activity in inclusion bodies showed the existence of properly folded asparaginase tetramers. Expression temperature affected the properties of asparaginase inclusion bodies. Inclusion bodies expressed at higher temperatures were characterized by higher biological activity and less amyloid content as evident by Thioflavin T binding and Fourier Transform Infrared (FTIR) spectroscopy. Complex kinetics of proteinase K digestion of asparaginase inclusion bodies expressed at higher temperatures indicate higher extent of conformational heterogeneity in these aggregates.

## Introduction

High level expression of recombinant proteins in *Escherichia coli* often results in the formation of inclusion bodies (Williams et al., 1982; Freedman and Wetzel, 1992; Chrunyk et al., 1993). Formation of inclusion bodies poses a major bottleneck in high-throughput recombinant protein production and necessitates the optimization of appropriate solubilization and refolding strategies (Singh and Panda, 2005; Singh et al., 2015a,b). Use of spectroscopic techniques like Fourier Transform Infrared (FTIR) spectroscopy and Nuclear Magnetic Resonance (NMR) spectroscopy has revealed the fine structure of inclusion body aggregates (Wang, 2009). In contrast to the conventional view of inclusion bodies as irreversible aggregates, it has been reported that they are dynamic, reversible structures (Carrio and Villaverde, 2001, 2002). Inclusion bodies have been demonstrated to have amyloid like properties and are shown to contain cross-beta structures (Carrio et al., 2005; Morell et al., 2008; Wang et al., 2008; de Groot et al., 2009; Elia et al., 2017). There are several reports in support of presence of native-like secondary structures in inclusion bodies (Oberg et al., 1994; Przybycien et al., 1994; Umetsu et al., 2004; Ventura and Villaverde, 2006). The presence of enzyme activity, and thus, native-like tertiary structure in inclusion bodies has also been reported (Garcia-Fruitos et al., 2005, 2007; Peternel et al., 2008; Peternel and Komel, 2010; Flores et al., 2019). This has opened a door to several novel biotechnological applications of inclusion body aggregates (Roessl et al., 2010; Carvajal et al., 2011; Gatti-Lafranconi et al., 2011; Vazquez et al., 2012; Villaverde et al., 2012; Krauss et al., 2017; de Marco et al., 2019).

Inclusion bodies with significant biological activity are known as non-classical inclusion bodies (Jevsevar et al., 2005; Garcia-Fruitos et al., 2012) and are characterized by loose molecular arrangement of protein molecules which can be solubilized at low concentration of denaturants like urea (Upadhyay et al., 2012). Presence of amyloid-like structure and biological activity supports the existence of multiple protein conformations inside these aggregates. Non-classical inclusion bodies have been reported to be susceptible to proteolysis by non-specific proteases and are known to be easily solubilized with low concentrations of denaturants (Upadhyay et al., 2012). Structural analysis of non-classical inclusion bodies has revealed that they have less proportion of beta content suggesting less proportion of protein molecules involved in amyloid formation (Ami et al., 2005).

Proportion of native molecules in this heterogeneous environment depends upon the conditions used during production of recombinant protein (Ami et al., 2005; de Groot and Ventura, 2006; Villaverde et al., 2012). Temperature used during expression is one of such physical parameters which can modulate the structural quality of resulting inclusion bodies. There are a number of reports supporting the view that decreasing the expression temperature enhances the quality of inclusion bodies in terms of biological activity and use of low expression temperature can favor the formation of non-classical inclusion bodies (Ami et al., 2005; Jevsevar et al., 2005; Peternel et al., 2008). Thus, it is important to study the effect of temperature on quality of inclusion bodies and to correlate it with their structural properties.

Studies concerning active inclusion bodies of oligomeric proteins or enzymes are limited, beta lactamase (Przybycien et al., 1994) and beta galactosidase (Worrall and Goss, 1989) being the only well studied cases. *E. coli* L-asparaginase II, which is a tetramer in its native state, was used as a model protein (Swain et al., 1993). Asparaginase was expressed as inclusion bodies at different temperatures. Inclusion bodies were purified and characterized using various techniques. The aim was to study the effect of expression temperature on the quality of inclusion bodies in terms of biological activity, presence of amyloid-like structures, secondary structural content and arrangement of protein molecules inside inclusion bodies. The outcomes of the study were correlated to establish a structure-function relationship of inclusion bodies expressed at different temperatures.

## Materials and Methods

### Chemicals and Reagents

Components for culture media, tryptone, and yeast extract were purchased from Difco Laboratories, India. Glycine, phenylmethylsulfonyl fluoride (PMSF), isopropyl β-D-1-thiogalactopyranoside (IPTG), Tris buffer and sodium dodecyl sulfate (SDS) were from Amresco, United States. Glucose and NaCl were purchased from Qualigen, India. Low molecular weight marker for SDS-PAGE was from GE Healthcare, United States. Dithiothreitol (DTT), acrylamide, bis-acrylamide, urea, ammonium persulfate (APS) and Proteinase K were purchased from Sigma-Aldrich, United States. Bromophenol blue, Tetramethylethylenediamine (TEMED), and Ethylenediaminetetraacetic acid (EDTA) were procured from BIO-RAD, United States.

### Expression of Asparaginase II and Purification of Inclusion Bodies

*Escherichia coli* BL21 (DE3) cells expressing *E. coli* asparaginase II were grown in modified Luria-Bertani media (containing 5 g/l glucose) in shaker flask culture in orbital shaker (200 rpm) at 37°C till the optical density at 600 nm (OD_600 *nm*_) reached 0.8. Cells were then transferred to orbital shakers maintained at different temperatures (20, 30, 37, or 42°C) before induction and were induced with IPTG (final concentration 1 mM). Cells were induced for 3 h. Cells were harvested by centrifugation at 10,000 × g for 10 min (4°C). Small amounts of cell pellets were resuspended in buffer 1 (50 mM Tris-HCl, 1 mM EDTA, 1 mM PMSF, pH 8.5) to form suspensions of equal OD_600 *nm*_. Resuspended cells were lysed using sonication for 5 cycles of 1 min each (with short pulses of 1 s followed by a gap of 1 s) with 1 min gap between the cycles (sonicator was operated at 30% amplitude), followed by centrifugation at 12,000 × g for 20 min at 4°C. Supernatants and pellets were separated. Pellets were resuspended in buffer 1 to achieve volumes equal to the volumes of supernatants. Equal volumes of respective pellets and supernatants were analyzed on SDS-PAGE gel. The fractions of expressed asparaginase in the respective lanes were determined densitometrically using ImageJ image processing package.

Rest of the *E. coli* cells expressing asparaginase at different temperatures were sonicated and centrifuged to isolate pure inclusion bodies as mentioned previously (Upadhyay et al., 2014). Multiple sonication and washing steps yielded purified inclusion bodies. Inclusion bodies purified from equal amounts of cells were solubilized in 2% SDS and analyzed on SDS-PAGE gel. The percentage of asparaginase in each inclusion body preparation (purity of inclusion bodies) was determined by densitometry using ImageJ package. Total amount of protein in each of the purified inclusion body preparations was determined using Micro BCA protein assay kit (Thermo Scientific). Final asparaginase concentration in inclusion bodies was determined from total protein concentration and purity of inclusion bodies. Isolated inclusion bodies were kept at 4°C till further use for experiments. SDS-PAGE was performed on a slab gel with 5% polyacrylamide stacking gel and 12% polyacrylamide running gel.

### Asparaginase Activity Assay

Asparaginase catalyzes the hydrolysis of L-asparagine into L-aspartate and ammonia. Activity assay for asparaginase is based on colorimetric detection (at 450 nm) of released ammonia by Nessler’s reagent and was performed as reported earlier (Wriston, 1985; Charbonneau et al., 2017). Reaction mixture for the assay consisted of 7 mM L-asparagine and 50 mM Tris-HCl buffer at pH 8.5 (Pre-equilibrated at 37°C) and purified asparaginase inclusion bodies (10 μg/ml final asparaginase concentration). Inclusion bodies were freshly centrifuged at 12,000 g and the pellet was resuspended to the desired concentration prior to the assay. The reaction was carried out for 10 min at 37°C. 1.5 M trichloroacetic acid was added to stop the reaction. Reaction mixture was centrifuged at 12,000 g for 15 min and Nessler’s reagent was added and ammonia released was determined using ammonium sulfate as standard (Charbonneau et al., 2017).

### Thioflavin T Binding Assay

Binding of inclusion bodies to Thioflavin T (ThT) was carried out to study amyloid nature of asparaginase inclusion bodies expressed at different temperatures. Briefly, inclusion bodies expressed at different temperatures were diluted with 50 mM Tris-HCl buffer (pH 8.5) to form suspensions of equal optical density (measured at 350 nm, OD_350 *nm*_ = 0.3). Inclusion bodies suspensions were incubated with 50 μM ThT for 15 min. Fluorescence spectra were recorded on Cary Eclipse spectrofluorimeter (Varian, United States). Samples were excited at 440 nm and emission spectra were recorded in the range of 460–600 nm with excitation and emission slit widths set to 5 nm. Data were smoothed using 5-point Savitzky-Golay smoothing. ThT without protein was taken as negative control. Difference spectra (after deducting spectra of the above negative controls) were used for analysis. An average of three independent spectra was taken for final analysis.

### Solubilization of Asparaginase Inclusion Bodies in Urea

Asparaginase inclusion bodies (100 μl) expressed at different temperatures were diluted to equal optical density and were solubilized in 900 μl of 50 mM Tris-HCl buffer (pH 8.5) containing different concentrations of urea (from 0 to 8 M). Samples were incubated at room temperature for 3 h for solubilization. Samples were vortexed and optical density at 350 nm (OD_350 *nm*_) was measured for each sample. Urea solubility profiles for each type of inclusion bodies were obtained by plotting OD_350 *nm*_ against urea concentration.

### Proteinase K Digestion

Susceptibilities to digestion with proteinase K for asparaginase inclusion bodies expressed at different temperatures were determined. Inclusion bodies were diluted to equal optical densities at 350 nm (OD_350 *nm*_) with Tris-HCl buffer (pH 8.5) (final volume 980 μl). Proteolytic digestion was initiated by adding 20 μl of proteinase K solution (0.3 mg/ml). Reaction was monitored by measuring OD_350 *nm*_ for 1 h. All the measurements were made on UV-2450 spectrophotometer (Shimadzu, Japan) at 37°C maintained by temperature controller attached to the spectrophotometer.

### ATR-FTIR Spectroscopy of Asparaginase Inclusion Bodies

Asparaginase inclusion bodies expressed at different temperatures were dialyzed against 20 mM phosphate buffer prepared in D_2_O (pD 7.5). Phosphate buffer without inclusion bodies was used as blank. Wet samples were applied directly on Bruker Tensor II ATR-FTIR spectrometer (Bruker Corporation, United States). Sixty-four interferograms were recorded in range of 1,200–2,000 cm^–1^ and were averaged for data analysis. Data points were subjected to Fourier self-deconvolution after deduction of blank spectra and linear baseline subtraction. Second derivatives of deconvoluted amide I region spectra were determined. Secondary derivative data were analyzed to find out the frequencies at which different spectral components were located. These frequencies were used for assignment of secondary structural contents in inclusion bodies. After detection of major bands, reiterative curve fitting was performed on the non-deconvoluted spectra using OriginPro 8.6. Area under the relevant curves was determined to quantitate secondary structures present in inclusion bodies.

## Results

### *E. coli* Asparaginase II Expression at Different Temperatures

**FIGURE 1:**
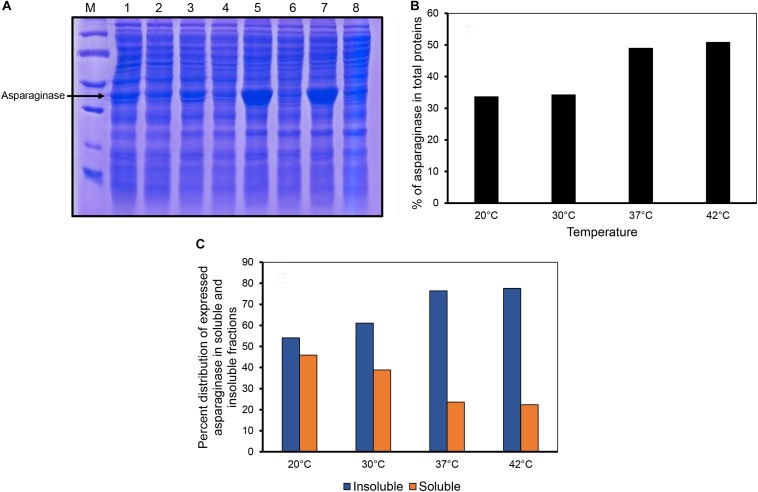
Expression of asparaginase at different induction temperatures. **(A)** SDS-PAGE analysis of solubility profiles of asparaginase expressed at different induction temperatures. Lane M, molecular weight markers (97, 66, 45, 30, and 20.1 kDa). Lanes 1, 3, 5, and 7 are insoluble fractions obtained at 20, 30, 37, and 42°C, respectively. Lanes 2, 4, 6, and 8 are the soluble fractions obtained at 20, 30, 37, and 42°C, respectively. **(B)** Percentage of expressed asparaginase with respect to the total cellular proteins. **(C)** Distribution of expressed asparaginase into soluble and insoluble (inclusion bodies) fractions.

*E. coli* asparaginase II was expressed by inducing the culture at different temperatures. The proportion of asparaginase expressed relative to the total cellular proteins increased with the induction temperature used (Figures 1A,B). Densitometric analysis of the SDS-PAGE gel revealed that the percentage of expressed asparaginase in insoluble fraction increased with expression temperature as shown in Figure 1C. Interestingly, even at low induction temperatures of 20 and 30°C, asparaginase aggregated in inclusion bodies, showing the aggregation prone nature of the protein.

### Biological Activity in Inclusion Bodies of Asparaginase Induced at Different Temperatures

**FIGURE 2:**
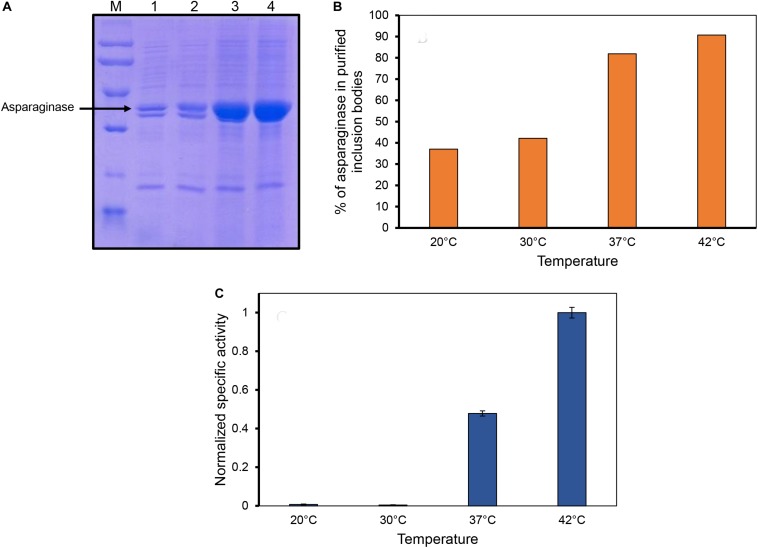
Inclusion body purification and determination of asparaginase activity in purified inclusion bodies. **(A)** SDS-PAGE analysis of purified inclusion bodies expressed at different temperatures. Lane M, low molecular weight marker (97, 66, 45, 30, and 20.1 kDa); Lane 2, lane 3, lane 4, and lane 5 are asparaginase inclusion bodies expressed at 20, 30, 37, and 42°C, respectively. **(B)** Percentage of asparaginase in purified asparaginase inclusion bodies. **(C)** Normalized specific activities of asparaginase inclusion bodies expressed at different temperatures. Error bars represent standard deviations from two independent experiments (*N* = 2).

Inclusion bodies were isolated from the cells induced at different temperatures by sonication and multiple washing steps to achieve high purity levels (Figures 2A,B). As the expression levels of asparaginase induced at lower temperatures (20 and 30°C) were low, less amounts of purified inclusion bodies were produced in these cases. Purified inclusion bodies were checked for the presence of biological activity. Asparaginase is an amidohydrolase which catalyzes the hydrolysis of L-asparagine into aspartate and ammonia. The formation of product was checked by photometric detection of ammonia with Nessler’s reagent as mentioned in methods section. Biological activity of asparaginase inclusion bodies was found to have a direct relationship with temperature of induction (Figure 2C). Inclusion bodies expressed at low temperatures were found to have lower activity in comparison to those expressed at higher temperatures. Inclusion bodies expressed at 42°C were found to be most active. The specific activities determined for different inclusion bodies are shown in Figure 2C. This was an unprecedented observation as the earlier reports dealing with the effect of expression temperature on the quality of inclusion bodies suggest that lower expression temperatures result in inclusion bodies with higher biological activity (Peternel et al., 2008). To establish a structure-activity relationship of the inclusion bodies, structural analysis was carried out.

### Asparaginase Inclusion Bodies Have Amyloid-Like Structure

ATR-FTIR spectroscopy was used to determine secondary structure elements in asparaginase inclusion bodies induced at the four temperatures. Briefly, blank subtracted FTIR spectra from amide I region (1,700–1,600 cm^–1^) were Fourier self-deconvoluted and the second derivatives were plotted against wavenumber. The second derivative spectra were used to identify major bands, which were assigned to the respective secondary structures based on the established practices (Goormaghtigh et al., 1994; Barth and Zscherp, 2002; Zandomeneghi et al., 2004; Li et al., 2019; Table 1). Peak analysis by iterative curve fitting process was used to obtain individual Gaussian components. Area under these component curves were used to determine protein secondary structure composition of each inclusion body preparation.

**TABLE 1:** Assignment of amide I band positions to secondary structure components used in the study (Goormaghtigh et al., 1994; Zandomeneghi et al., 2004; Li et al., 2019).

Secondary structure	Band Position in D_2_O (cm^–1^)		
	Average	Extreme	
α-helix	1,652	1,642–1,660	
β-sheet	1,630	1,610–1,640	
		Native protein	Amyloid aggregates
		1,630–1,640	1,610–1,630
Disordered	1,645	1,639–1,654	
β-turn	1,671	1,653–1,691	
β-sheet	1,679	1,672–1,694	

**FIGURE 3:**
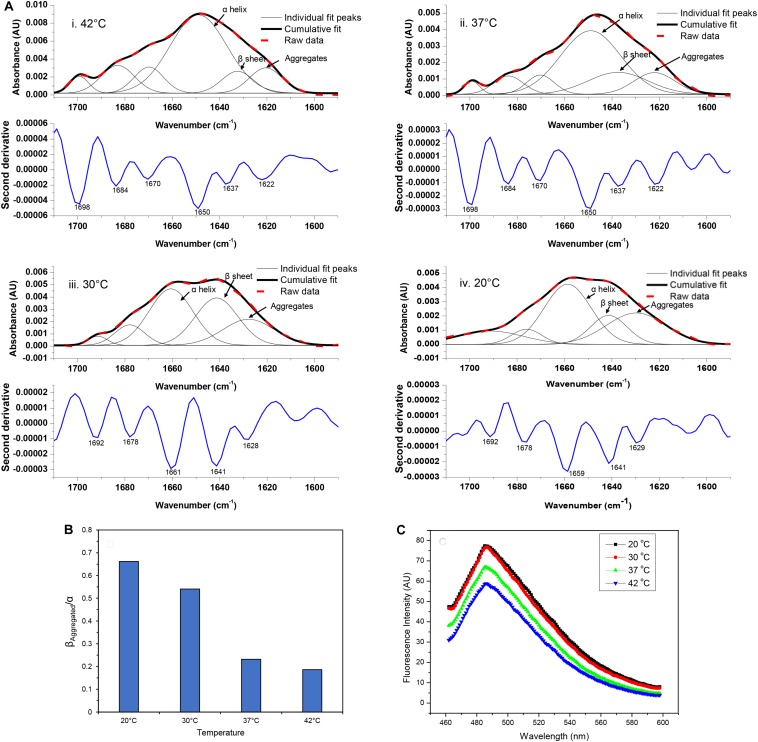
Amyloid content in asparaginase inclusion bodies. **(A)** Amide I FTIR spectra of asparaginase inclusion bodies induced at different temperatures (i. 42°C, ii. 37°C, iii. 30°C, and iv. 20°C). Second derivatives of Fourier self-deconvoluted spectra (shown in blue lines, labeled minima values are in cm^– 1^) were used to identify and assign major structural components. With help of the identified peaks, curve fitting of amide I band spectra was performed to obtain individual curves for the secondary structure components. Raw data (bold red dashed lines), cumulative fits (bold black lines), and individual peak components (black lines) are shown above the respective second derivatives. The component bands assigned as alpha-helix (α helix), beta sheet in native structure (β sheet), and beta structure present in amyloid aggregates (Aggregates) are pointed out with black arrows. **(B)** Ratio of beta structure present in amyloid aggregates and alpha helical component (β_*Aggregated*_/α) was used as a measure of amyloid content present in asparaginase inclusion bodies expressed at different temperatures. **(C)** Comparison of amyloid content in asparaginase inclusion bodies expressed at different temperatures using Thioflavin T binding.

The FTIR spectrum of asparaginase inclusion bodies induced at 42°C showed major band at 1,650 cm^–1^, which was assigned as alpha helix (Figure 3Ai). The lower components of extended beta structures at 1,637 and 1,622 cm^–1^ were assigned as beta sheets in globular protein and beta structures in protein aggregates, respectively. The three bands in higher frequency region at 1,698, 1,684, and 1,670 cm^–1^ were assigned as beta turns. The area under the curve corresponding to the band at 1,622 cm^–1^ was used to quantify amyloid aggregation in inclusion bodies. Analysis revealed that these inclusion bodies had around 10% of aggregated beta structures. Remarkably, the inclusion bodies contained around 55% alpha helical structure, which is close to 52% alpha helical structure in native asparaginase as determined by X-ray crystal structure (Swain et al., 1993). The FTIR spectrum of inclusion bodies expressed at 37°C had similar profile with slightly increased aggregated beta structures (12%) and with reduced alpha helical structure (52%) (Figure 3Aii).

The visual comparison of the Amide I region of FTIR spectra of inclusion bodies expressed at lower temperatures (20 and 30°C) and higher temperatures (37 and 42°C) show the decrease in alpha helical and increase in beta content as the temperature of induction is reduced (Figure 3A). In inclusion bodies induced at lower temperatures, the bands in region 1,659–1,661 cm^–1^ were assigned as alpha helices. The band at 1,641 cm^–1^ was assigned as beta structure in native protein while the bands in region 1,628–1,629 cm^–1^ were assigned as intermolecular beta structures present in protein aggregates (Figures 3Aiii,iv). There was a clear decrease in the alpha helical structure in these inclusion bodies as compared to those induced at higher temperatures (Table 2). Furthermore, the shift in helical band to higher frequency (∼1,660 cm^–1^ vs. 1,650 cm^–1^) is presumably a consequence of weakened alpha helical structures in these inclusion bodies as longer and stronger alpha helices are known to absorb at lower frequencies (Dousseau and Pezolet, 1990; Gorne-Tschelnokow et al., 1993).

**TABLE 2:** Percentage of alpha helical structure and beta sheet component from amyloid aggregates in asparaginase inclusion bodies expressed at different temperatures.

Induction temperature	Percentage of alpha helix (α)	Percentage of beta content found in amyloid aggregates (β_*Aggregated*_)
20°C	39.8	26.35
30°C	37.34	20.18
37°C	52.29	12.13
42°C	55.64	10.35

Amorphous protein aggregates usually absorb at around 1,620 cm^–1^ (Shivu et al., 2013). In contrast, amyloid aggregates of the same protein results in a band shifted to a higher wavenumber of around 1,628 cm^–1^ (Shivu et al., 2013). Such shift has been used earlier to study progression of amyloid aggregation (Yushchenko et al., 2018) and as a tool to purify and characterize amyloid aggregates (Zurdo et al., 2001). The reduction of induction temperatures resulted in the shift of the “aggregates” band from 1,622 to 1,628 cm^–1^, suggesting presence of more “amyloid-like” aggregation in inclusion bodies induced at 20 and 30°C (Figure 3A). This observation, along with the increased percentage of intermolecular beta structure (26% in inclusion bodies expressed at 20°C), showed that amyloid content in asparaginase inclusion bodies increased with decrease in induction temperature (Table 2). The ratio of intermolecular beta (β_*Aggregated*_) and alpha helical (α) components (β_*Aggregated*_/α) has been used previously as a measure of amyloid content (Klementieva et al., 2017). As shown in Figure 3B, β_*Aggregated*_/α decreased with increasing induction temperature, confirming that asparaginase inclusion bodies induced at lower temperatures have higher amyloid content.

In order to compare their amyloid-like nature, asparaginase inclusion bodies expressed at different temperatures were subjected to ThT binding (Figure 3C). Inclusion bodies showed binding to ThT dye and exhibited the fluorescence maxima at 485 nm, a characteristic of amyloid aggregates (Ban et al., 2003). This suggested the presence of cross-beta structures in asparaginase inclusion bodies. Fluorescence intensities indicated that inclusion bodies expressed at lower temperatures have stronger amyloid character (Figure 3C). These results were in agreement with the FTIR results presented above and showed that the amyloid content present in inclusion bodies decreased with increasing induction temperature.

### Conformational Heterogeneity in Asparaginase Inclusion Bodies

**FIGURE 4:**
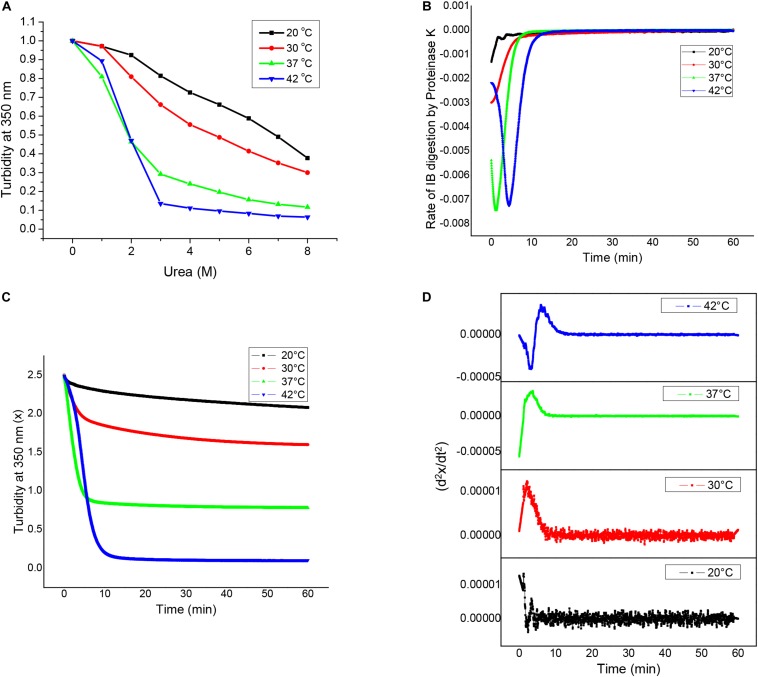
Structural analyses of asparaginase inclusion bodies as a function of induction temperature. **(A)** Urea solubilization profiles for asparaginase inclusion bodies expressed at different temperatures. **(B)** Proteinase K digestion profiles of asparaginase inclusion bodies expressed at different temperatures. **(C)** Rates of inclusion body digestion with respect to time for different asparaginase inclusion bodies. These rates were obtained by differentiating the data represented in **(B)** with respect to time. **(D)** Double differential of turbidity at 350 nm with respect to time (d^2^x/dt^2^) for asparaginase inclusion bodies.

Inclusion bodies which display considerable amount of activity, also known as non-classical inclusion bodies, are easily solubilized in presence of denaturants. Urea denaturation profiles for asparaginase inclusion bodies expressed at different temperatures were obtained by solubilizing inclusion bodies at different urea concentration and monitoring the extent of solubilization by making optical density measurements at 350 nm (OD_350_). Figure 4A shows urea solubility profiles for different inclusion bodies. It was observed that higher expression temperatures resulted in inclusion bodies which were easy to be solubilized by urea. Inclusion bodies expressed at 42°C almost completely solubilized even at urea concentrations as low as 3 M. In contrast, inclusion bodies expressed at 20°C were resistant to urea solubilization and were not fully solubilized even in 8 M urea. Although these observations were contradictory to most of the existing reports on the subject, they established the non-classical nature of asparaginase inclusion bodies expressed at higher temperatures.

Susceptibility for proteinase K has been used to characterize bacterial inclusion body aggregates as well as amyloid fibrils. To characterize asparaginase inclusion bodies expressed at different temperatures, they were subjected to proteinase K treatment. For this, inclusion body suspensions with equal optical density at 350 nm (OD_350 *nm*_) were treated with proteinase K and their susceptibilities for digestion were measured by monitoring OD_350 *nm*_ with time. All four types of inclusion bodies were found to be susceptible to proteinase K digestion, though the extents varied markedly. It was observed that asparaginase inclusion bodies expressed at higher temperatures (37 and 42°C) were more susceptible to digestion in comparison to those expressed at low temperatures (20 and 30°C). Figure 4B shows the Proteinase K digestion profiles for different inclusion bodies. It was observed that there was a direct relation between expression temperature and susceptibility to proteinase K digestion. This showed that the molecular arrangement of inclusion bodies expressed at low temperatures was more compact than that of inclusion bodies expressed at higher temperatures. As amyloid structures are known to be compact and resistant to proteolysis, these results along with ThT binding results suggest that inclusion bodies expressed at lower temperatures have more amyloid-like content. Inclusion bodies expressed at induction temperature of 42°C were digested completely by proteinase K, while those expressed at lower induction temperature were resistant to digestion showing increase in proportion of protease resistant structures inside inclusion bodies.

To get an insight into the differences in kinetics of proteinase K digestion for different inclusion bodies, rate of inclusion body digestion was plotted against time as shown in Figure 4C. Kinetics data showed considerable differences in the way proteinase K acted on inclusion bodies expressed at different temperatures. Digestion of inclusion bodies expressed at 20°C showed simplest kinetics with the degradation rate decreasing with time. Inclusion bodies expressed using induction temperature of 30°C also showed a decrease in the reaction rate with time, but the decrease in the digestion rate in this case was found to follow a sigmoidal pattern. Digestion of inclusion bodies expressed at higher temperatures (37 and 42°C) showed more complex kinetics, with the rate first increasing and then decreasing with time. In both the cases, the decrease in the rate of digestion showed a sigmoidal pattern. The kinetics of proteinase K digestion for inclusion bodies expressed at 42°C showed an increase in the reaction rate, followed by its subsequent decline.

To confirm these observations, a double differential of OD_350 *nm*_ with respect to time was plotted against time as shown in Figure 4D. The presence of bell-shaped curves in cases of inclusion bodies expressed at 30 and 37°C confirmed that the decrease in rate of digestion followed a sigmoid function. The complex kinetics of digestion of inclusion bodies expressed at induction temperature of 42°C was also confirmed as the double differential. Figures 4C,D show that the complexity of the kinetics of inclusion body digestion by proteinase K increases with increase in the expression temperature.

## Discussion

Recent reports on the fine structure of bacterial inclusion bodies suggest heterogeneous nature of inclusion bodies (Carrio and Villaverde, 2001, 2002; Carrio et al., 2005; Morell et al., 2008; Wang et al., 2008; de Groot et al., 2009; Wang, 2009). Furthermore, non-classical inclusion bodies have been shown to have biological activity. Recently, presence of activity has also been demonstrated in stress granules formed in heat shocked yeast cells, making studies on active protein aggregates more relevant (Wallace et al., 2015). In the present study, the effect of expression temperature on formation of non-classical inclusion bodies of an oligomeric protein, *E. coli* L-asparaginase II has been studied. Complex kinetics of digestion of asparaginase inclusion bodies expressed at higher temperatures suggests the presence of multiple conformers in these aggregates having different susceptibilities for proteinase K digestion. Inclusion body aggregates are thought to be consisting of a network made up of amyloid-like structures in which protein molecules having unfolded, partially folded and even native conformations are trapped. This conformational heterogeneity was found to increase with increase in expression temperature. ThT binding and ATR-FTIR spectroscopy data suggested reduced amyloid-like content in inclusion bodies expressed at high temperatures. Presence of high proportion of protein molecules with native conformation as shown by the activity data suggest that these aggregates consisted of multitude of conformers including the native ones. This was in agreement with the results obtained from proteinase K digestion experiments.

Observations from the present study reveal the structural details of the inclusion body aggregates and provide valuable insight into the mechanism of their formation. Expression levels at high temperatures are high due to increased rate of translation. Furthermore, with increasing expression temperature the fraction of total expressed protein going into inclusion bodies also increases. Inclusion bodies expressed at higher temperatures tend to be structurally heterogeneous with a framework of amyloid like structures containing structurally diverse protein molecules embedded into them. High concentration of expressed protein molecules in bacterial cytosol might be an important factor causing these temperature dependent differences. At low temperatures the protein concentration is less, thus only those protein molecules which have conformations having high propensity to get into amyloid aggregation participate in inclusion body formation. Therefore these inclusion bodies are more homogeneous in nature and have high amyloid content. As expression temperature is increased, the protein concentration increases which results in macromolecular crowding, enhancing the chances of native asparaginase tetramers as well as other conformers getting trapped in inclusion bodies. This view is supported by a recent report on *in vitro* aggregation of β_2_-microglobulin, demonstrating the formation of amorphous aggregates with decreased amyloid-like properties when high protein concentration was used for aggregation (Adachi et al., 2015).

Previous reports have shown that low expression temperatures favor the production of non-classical inclusion bodies (Ami et al., 2005; Jevsevar et al., 2005; Peternel et al., 2008). This study contradicts this view and shows that increasing expression temperature can also result in formation of non-classical inclusion bodies. Non-classical inclusion bodies offer a wide range of biotechnological applications, making it necessary to standardize expression conditions to manipulate their quality. In contrast to the generalized conditions considered to promote formation of non-classical inclusion bodies (such as use of low temperature during induced expression), the results here suggest that these conditions depend upon the nature of protein and thus should be optimized individually.

## Data Availability Statement

The raw data supporting the conclusions of this article will be made available by the authors, without undue reservation, to any qualified researcher.

## Author Contributions

AnS, VU, and AP designed the experiments. AnS, VU, and AkS performed the experiments. AnS and AP prepared the manuscript. All authors read and approved the final manuscript.

## Conflict of Interest

The authors declare that the research was conducted in the absence of any commercial or financial relationships that could be construed as a potential conflict of interest.
